# Evidence-based Chinese Medicine Clinical Practice Guideline for Stroke in Hong Kong

**DOI:** 10.1186/s13020-020-00397-9

**Published:** 2020-11-03

**Authors:** Linda L. D. Zhong, Wai Kun, Nannan Shi, Tat Chi Ziea, Bacon F. L. Ng, Ying Gao, Zhaoxiang Bian, Aiping Lu

**Affiliations:** 1 Hong Kong Chinese Medicine Clinical Study Centre, Hong Kong Baptist University, Kowloon Tong, Hong Kong SAR, China; 2 School of Chinese Medicine, Hong Kong Baptist University, 4F, Jockey Club School of Chinese Medicine Building, 7 Baptist University Road, Kowloon Tong, Hong Kong SAR, China; 3 Institute of Basic Research in Clinical Medicine, China Academy of Chinese Medical Sciences, Beijing, China; 4 Chinese Medicine Department, Hong Kong Hospital Authority, Lok Fu, Hong Kong; 5 Department of Neurology, Dongzhimen Hospital, Beijing University of Chinese Medicine, Beijing, China

**Keywords:** Chinese medicine, Clinical practice guideline, Stroke, Acupuncture, Evidence-based medicine

## Abstract

**Background:**

Stroke in Chinese Medicine (CM) includes the concepts of ischemic and hemorrhagic strokes from Western Medicine and is a common disease in Hong Kong. This clinical practice guideline (CPG) aims to evaluate and demonstrate CM treatment options for stroke, provide guideline for local CM practice, and act as a reference for decision makers on drafting CM related health policies.

**Methods:**

Based on the principle of multidisciplinary integration and evidence-based medicine, a steering committee oversaw the CPG development process in accordance with a published protocol. Clinical questions and evidences were identified, appraised, and synthesised through systematic literature reviews, text mining, and two rounds of Delphi surveys with a multidisciplinary panel of experts.

**Results:**

In this CPG, we defined stroke from the perspectives of both CM and Western Medicine, reported corresponding CM treatment options, and carried out evaluation based on levels of evidence and grade of recommendation. Suggested CM interventions include herbal medicine treatment based on pattern differentiation, acupuncture treatment, and nursing care.

**Conclusion:**

The target population is Hong Kong stroke patients with prodrome or sequela stage. This CPG is intended to help standardizing CM clinical practice and enhancing efficiency of clinical service in Hong Kong.

## Background

Stroke is a neurological disease induced by cerebral infarction, intracerebral hemorrhage, or subarachnoid hemorrhage. It is a major cause of disability and death in the world. Concept of stroke in CM includes all the types of the above definition in Western Medicine. In 1986, the stroke professional group of internal medicine branch from China Association of Chinese Medicine (CACM) developed the “Criterion for CM Diagnosis and Efficacy of Stroke” [1]. From 1991–1996, a scientific research group conducted a multi-center clinical investigation of CM syndromes of stroke. Based on the results, the collaborative group of cerebral emergency from the State Administration of Traditional Chinese Medicine of the People’s Republic of China developed the second version of the “Criteria for CM Syndromes and Efficacy of Stroke” [2] and “Standards of Syndrome-differentiated Diagnosis in Stroke (Trial) [3]. From 1996–2011, China completed the International Cooperation Project “The Traditional Chinese Medicine Rehabilitation of Cerebrovascular Disease” of World Health Organization and developed different versions of CPGs [4–6]. However, these CPGs were developed on the basis individual experience or expert’s consensus and not suitable for Hong Kong’s real practice environment.

In Hong Kong there is no CMCPG published by any authority on guiding clinical practice. The main differences in CPG requirements between Hong Kong and mainland China reflect the emphasis on evidence-based medicine and the regulatory system of CM in Hong Kong that, for example, prevents Chinese Medicine practitioners (CMPs) from prescribing Western medicine and ordering laboratory tests. Limitations of the CACM CPGs also include a lack of bilingual publications and tailoring of the recommendations to optimize dissemination and implementation in the Hong Kong setting. With the increasing number of patients seeking CM services, there is an urgent need to develop localized CM CPGs to standardize CM practice and promote evidence-based CM practice. In response, the Hospital Authority in Hong Kong launched the project for development of CM CPGs in 2013 and commissioned Hong Kong Baptist University to complete the CPG development process. The purpose is to evaluate existed CM treatment options for stroke and provide guidance on (1) the classification of CM patterns; (2) Chinese herb treatments; (3) acupuncture and moxibustion treatments; and (4) an evidence-based CM regimen for the management of stroke in Hong Kong.

## Methods

A protocol outlining the CPG process has been published [7]. Guideline development was informed by national and international procedures and standards [8–10] under the direction of a steering committee and a multidisciplinary panel of experts. Details of the three phases (preparation, development, and finalisation) are outlined in Additional file 1: Appendix S1. The research was approved by Hong Kong Baptist University Research Ethics Committee.

### Establishment of steering committee and panel

The steering committee consisted of three specialists and two methodology experts. All of the committee members had CM CPG research and development experience. The role of the steering committee was to provide advice and conduct an external review of the contents and methodology.

The multidisciplinary panel included all research members, health care policy makers, experts from medicine, methodology and literature, patient representatives, and local CM practitioners. The multidisciplinary panel’s responsibility was to conduct the study.

### Systematic review and evaluation

We conducted a systematic search of clinical research regarding CM treatments for stroke by following databases: Cochrane DSR, ACP journal Club, DARE, CCTR, CMR, HTA, And NHSEED; EMBASE (1980–2014.1), EMBASE Classic (1947–1979); Ovid MEDLINE(R) (1950–2014.1); Ovid OLDMEDLINE(R) 1948–1965; China Journals Full-text Database (1994–2014.1); and CNKI (1979–2014.1). All relevant published studies within the databases, either in Chinese or English were identified. The following search terms were used: “stroke” AND “Chinese herb” OR “acupuncture” OR “massage” OR “Chinese medicine” OR “Traditional Chinese Medicine”, and a total of 4167 records for stroke were retrieved. Two reviewers independently evaluated and selected potentially relevant articles by screening the titles and abstracts of the retrieved records. The quality of the selected studies was appraised and ranked according to the standard level of evidence system by Prof. Liu [8–10].

### Identification of clinical questions from clinical evidences

Existing CM CPGs on stroke were identified through the electronic search and by manual retrievals of publications in Hong Kong and abroad. The Appraisal of Guidelines for Research & Evaluation (AGREE) II was used to evaluate their quality. Only one CPG had high methodology quality and was used as the basis of the clinical questions in this guideline (please refer to Additional file 2: Appendix S2).

### Identification of clinical questions from data mining

Clinical questions were further informed by searching for review articles reporting up-to-date clinical research and expert opinions on the management of stroke. To avoid repetition of clinical study and ensure efficiency, we identified clinical researches with CM treatment for stroke in CNKI (https://www.cnki.net/), the most comprehensive electronic medicine database in China. The time range was from 1979 to January 2014. The identified studies were downloaded from CNKI and their plain TXT data were transferred into another database (Microsoft SQL 2000) for further use.

### Delphi survey

Two rounds of Delphi surveys had been conducted. A questionnaire was formed based on the results generated from data mining process and high level of evidence collected. The questionnaire was designed as open-ended questions for collecting expert’s opinions on the priority and main contents from 56 CMPs. The first round of Delphi survey involved a total of 56 local CMPs with more than 5 years of clinical experience randomly selected from Hong Kong Registered Chinese Medicine Practitioners Association, which is the biggest professional organization of CM in Hong Kong.

The second round of Delphi survey involved the multidisciplinary panel of 20 experts in the area of stroke management. Newly nominated items and deleted items were listed in the second round questionnaire with close-ended questions. The purpose of this round of survey is to finalize the clinical questions.

### Process of assessment and consultation

Based on two rounds of Delphi surveys, the items of the guideline with strong consensus (more than 75%) were synthetically assessed and finalized in the CPG.

### Drafting guideline

A development group consisting of two CMPs and one methodologist, created a framework for the CPG and drafted the report.

## Results, recommendations and evidences

There have been recorded experiences in the treatment of stroke with CM since thousands of years ago. Early in “Inner Canon of Huangdi”, there were detail descriptions on stroke related symptoms [11]. Zhongjing Zhang from Han Dynasty thought the mechanism of the disease was emptiness of meridian vessel and direct strike of pathogenic wind [12]. Zhang classified the disease into collateral stroke, meridian stroke, bowel stroke and visceral stroke. During Song Dynasty, “Taipinghuiminhejijufang” mentioned the treatment of stroke fainting by both zhibao mimi-pills and suhexiang pills [13]. Medical practitioners during Jin and Yuan Dynasties emphasized possible causes of stroke included concept of “endogenous wind”. Wansu Liu used purgative method with dachengqi or sanhua decoction. In Ming Dynasty, Zhongzi Li classified the disease into wind stroke block pattern and collapse pattern. Qingren Wang applied buyang huanwu decoction for treatment of stroke and recorded 34 kind of syndromes of prodrome.

We searched 1412 abstracts and included 89 articles for full review. There were totally five seed CM CPGs for assessment and all the details were listed in Additional file 2: Appendix S2.

### Clinical questions

The following recommendations are accompanied by a rating that indicates the strength of the recommendation (A to C) and the level of the scientific evidence (I to V) (Additional file 3: Appendix S3). The symbol *, indicates when a statement is supported by the text mining results, and ★ indicates when a statement is supported by expert consensus.

#### What are the main clinical manifestations of stroke from a CM perspective?

Major clinical symptoms include fainting, hemiplegia, wry tongue, lallation or elinguid and hemianesthesia. The disease characteristics are acute onset and quick changes. It is a common disease occurs in elderly people. Other clinical signs include dizziness, double visual images, unsteady gait and bucking. Prodrome of wind stroke is closely related with stroke, it occurred more commonly in middle-aged people with diverse clinical signs, like paroxysmal vertigo, paroxysmal numbness in the affected body part(s), transient slurred speech or aphasia, transient hemiplegia, fainting, transient double visual, etc. Appropriate CM treatments can prevent or delay the onset of stroke from prodrome of wind stroke. Stroke can be mild or severe. The mild conditions are called attack of meridians and only limited to blood vessels or meridians without unconsciousness. The severe conditions are called attack of zang-fu organs and the patients present with unconsciousness [14, 15].

#### What are the CM patterns classification of stroke [16–20]

##### Prodrome of stroke

Pattern of ascendant hyperactivity of liver yang^*^: Paroxysmal vertigo, paroxysmal numbness in the affected area, transient slurred speech, transient hemiplegia, transient double visual, red face, headache with a distending sensation, red eyes and bitter taste, restlessness irritability, tremor of hands and feet, dark-yellow urine, red tongue with a thin and yellow coating or yellow and dry coating, wiry and rapid pulse.

Pattern of phlegm turbidity stagnation^*^: Paroxysmal vertigo, paroxysmal numbness in the affected area, transient slurred speech, transient hemiplegia, transient double visual, heavy-headedness, oppression in the chest with excessive phlegm, torpid intake and somnolence, heavy limbs, pale-pink or dark tongue, enlarged or tooth-marked, white or yellow greasy coating, wiry and slippery or soft and rapid pulse.

Pattern of phlegm qi deficiency and blood stasis^*^: paroxysmal vertigo, paroxysmal numbness in the affected area, transient slurred speech, transient hemiplegia, transient double visual, sallow face, palpitation and shout breath, spontaneous sweating and lack of strength, sloppy stool, dark tongue or ecchymosis or tooth-marked, white greasy coating, sunken and fine pulse.

Pattern of kidney deficiency and blood stasis^*^: paroxysmal vertigo, paroxysmal numbness in the affected area, transient slurred speech, transient hemiplegia, transient double visual, unsteadies walking, lack of strength, muscle spasm, lumbago, clear and profuse urine, delicate red tongue with scanty coating, fine pulse with weak chi or sunken and fine pulse.

##### Meridian stroke

Pattern of wind-phlegm obstructing the meridians^*^: Hemiplegia, deviation of the tongue and mouth, slurred speech or aphasia, numbness in the affected side, dizziness and blurred vision, sticky mouth with profuse sputum, dark tongue with white and greasy coating, wiry and slippery pulse.

Pattern of phlegm-heat the fu-organ^*^: Hemiplegia, deviation of the tongue and mouth, slurred speech or aphasia, numbness in the affected side, abdominal distension, dry stool or constipation, headache and blurred vision, expectoration and profuse sputum, dark-red tongue, yellow and greasy coating, wiry and slippery pulse or wiry, slippery and large pulse on the hemiplegia side.

Pattern of qi deficiency and blood stasis^*^: Hemiplegia, deviation of the tongue and mouth, slurred speech or aphasia, numbness in the affected side, bright pale complexion, shortness of breath and lassitude, angular salivation, spontaneous sweating, palpitation and sloppy stool, tumefaction of hands and feet, dark tongue with thin and white coating, tooth-marked, sunken and fine pulse.

Pattern of wind-stirring due to yin deficiency^*^: Hemiplegia, deviation of the tongue and mouth, slurred speech or aphasia, numbness in the affected side, dizziness and tinnitus, heat in the palms and soles, dry throat, red small tongue with no or scanty coating, wiry, fine and rapid pulse.

### Recommendations of Chinese medicine intervention

Treatment recommendations mostly apply to the management of stoke when a person is in a relatively stable condition. CM practitioners will need to consider potential risks associated with the concurrent use of pharmaceuticals.

Interventions for stroke are prescribed according to the principle of syndrome differentiation, and for Chinese herbal medicines according to the various stages of symptom changes.

#### Herbal medicine treatment

##### Circulating the blood to resolve stasis

This therapeutic approach was used in each period of stroke and has become very common among most of the experts. Besides, a number of clinical studies also supported the treatment method (Grading of recommendation: A, level of evidence: Ib**)** [21–24].

##### Tonifying qi and activating blood

This therapeutic approach is effective for the treatment of stroke. Buyinghuanwu Cecoction by Wang Qingren written in “Yilingaicuo” is its representative prescription (Grading of recommendation: A, level of evidence: Ib**)** [13].

#### Prodrome treatment

##### Pattern of ascendant hyperactivity of liver yang

Pathogenesis: Sudden hyperactivity of liver-yang leading to wind stirring and spasm of collaterals.

Principle: Pacify the liver to subdue yang.

Recommended recipe: Tian ma gou teng decoction modified (*Grading of recommendation: C, level of evidence: IV) [16].

##### Pattern of phlegm turbidity stagnation

Pathogenesis: Phlegm turbidity obstructing and stirring liver wind, wind-phlegm traveling upward to disturb and obstruct brain collaterals.

Principle: Extinguish wind and resolve phlegm.

Recommended recipe: Ban xia bai zhu tian ma decoction modified (*Grading of recommendation: C, level of evidence: IV) [19].

##### Pattern of phlegm qi deficiency and blood stasis

Pathogenesis: Deficiency of healthy qi and internal intention of stagnant blood leading to obstruction of the collaterals.

Principle: Tonifying qi and activating blood.

Recommended recipe: Bu yang huan wu decoction modified (*Grading of recommendation: C, level of evidence: IV) [19].

##### Pattern of kidney deficiency and blood stasis

Pathogenesis: Kidney yin deficiency failing to be nourishing liver and leading to endogenous wind, disharmony of qi and blood making stasis obstructing the collaterals.

Principle: Tonifying the kidney and activating blood.

Recommended recipe: Liu wei di huang wan decoction modified (*Grading of recommendation: C, level of evidence: IV) [18].

The elderly with above prodrome syndromes should be treated early to prevent the occurrence of stroke. General precautions include daily life, diet, climate, spirit and other aspects [25, 26] (*Grading of recommendation: C, level of evidence: IV).

##### Pattern of wind-phlegm obstructing the meridians

Pathogenesis: Liver wind mixed with phlegm, wind-phlegm obstructing the brain meridians,

Principle: To resolve phlegm, stop wind, circulate the blood and clear the meridians.

Recommended recipe: Hua tan tong luo decoction modified (*Grading of recommendation: C, level of evidence: IV) [20].

##### Pattern of phlegm-heat the fu-organ

Pathogenesis: Phlegm-heat obstructing the qi flows of fu-organs and wind-phlegm obstructing meridians.

Principle: To clear the heat in fu-organs and resolve phlegm.

Recommended recipe: Xing lou cheng qi decoction modified (*Grading of recommendation: C, level of evidence: IV) [27–29].

##### Pattern of qi deficiency and blood stasis

Pathogenesis: Deficiency of yuan-primordal qi and internal retention of stagnant blood leading to the obstruction of the blood flow and malnourishment of the meridians.

Principle: To supplement qi, circulate the blood and clear the meridians.

Recommended recipe: Bu yang huan wu decoction modified (“Yilingaicuo”) [30, 31] (*Grading of recommendation: C, level of evidence: IV).

##### Pattern of wind-stirring due to yin deficiency

Pathogenesis: Yin deficiency of the liver and kidney leading to internal stirring of wind-yang that obstructs meridians along with phlegm-turbidity and stagnant blood.

Principle: To nourish yin, inhibit yang, soothe the liver and stop wind.

Recommended recipe: Zhen gan xi feng decoction modified (*Grading of recommendation: C, level of evidence: IV) [16–19].

Yu yin xi feng decoction modified (*Grading of recommendation: C, level of evidence: IV) [16–18].

### Recommendations of Rehabilitation Treatment

#### Acupuncture

Acupuncture has been used to treat stroke for a long history and is still widely used in clinical practice. Once patient is under stable condition after acute phase, acupuncture should be carried out as soon as possible with appropriate manipulations and acupoints. Pregnant women should do acupuncture with caution. Treatment should be carried out by an experienced acupuncturist.

Principal points: Neiguan (PC 6) *, Sanyinjia (SP 6) *, Weizhong (BL 40) *, Baihui (GV 20), Shuigou (GV 26) *, Zusanli (ST 36) *, Quchi (LI 11) *, Waiguan (TE 5) *, Hegu (LI 4) *, Huantiao (GB 30) *, Yanglingquan (GB 34)* (Grading of recommendation: C, level of evidence: IV) [15].

Supplementary points: If upper limbs hemiplegia occurs, add Jianliao (TE 14) and Shousanli (LI 10); if lower limbs hemiplegia occurs, add Xuanzhong (GB 39) and Taichong (LR 3); if a deviation of the mouth or tongue occurs, add Dicang (ST 4) and Jiache (ST 6) [18].

Operation: Needling at Neiguan (PC 6) with a reducing method; needling at Shuigou (GV 26) with bird pecking method, in the degree of tearful eyes; needling at Sanyinjia (SP 6) with reinforce method; needling at Jiquan (HT 1) with lifting and thrusting, avoid artery and piercing needle. Make limbs with convulsive feeling by needling at Chize (LU 5) and Weizhong (BL 40) with lifting and thrusting [20].

#### Tuina (CM massage)

CM massage of the meridians and through the use of different kinds of therapeutic manipulations can increase the range of joints’ movements, relieve pain, and suppress spasms and passive movements. To promote the recovery of motor function based on acupoints massage, gradually improve the active movements of the affected limbs, such as upper limb expansion joint training and lower limb buckling joint training. Strong stimulation should be avoided on spasmed muscles and pay the same attention to the hemiplegia side of patients. Main therapeutic manipulations include kneading and pinching [32, 33] (Grading of recommendation: C, level of evidence: IV).

#### Fuming-washing therapy

This therapeutic approach is directly applying on the limbs by fuming and washing the diseased area with the vapor of a boiling decoction, which could have effects of relaxing sinews, activating collaterals, relieving pain, and reducing swelling [34] (Grading of recommendation: B, level of evidence: IIa).

### Elaborations of main complications treatment

#### Hiccupping

Hiccup is characterized by repetitive short “uh” throat sounds of uncertain duration. It is caused by adverse flow of stomach qi and the treatment is to harmonize the stomach and resolve the counter flow of qi [35].

##### Herbal medicine treatment

Constipation after stroke, yellow and greasy coating, frequent hiccups, recommended recipe is da cheng qi decoction modified (Grading of recommendation: B, level of evidence: IIb).

Pattern of deficiency of stomach yin with low sound hiccupping, red tongue with scanty coating, recommended recipe is yi wei decoction modified (Grading of recommendation: B, level of evidence: IIb) [18, 20].

##### Acupuncture

Principal points: Tiantu (CV 22), Danzhong (CV 17), Zhongwan (CV 12), Geshu (BL 17), Neiguan (PC 6), Zusanli (ST 36) (Grading of recommendation: C, level of evidence: IV) [33].

##### Dysphagia

Dysphagia after stroke is a common complication, swallowing water test can detect the majority of patients with dysphagia.

##### Herbal medicine treatment

Pattern of wind-phlegm obstructing the meridians with dysphagia, wheezy phlegm, uneasy expectoration, angular salivation, white greasy coating, wiry and slippery pulse, recommended recipe is jie yu pellet decoction modified (Grading of recommendation: C, level of evidence: IV) [16].

Pattern of qi deficiency and blood stasis with swallow complexion, shortness of breath and lassitude, hemiplegia, pale purple tongue, ecchymosis, rough and fine pulse, recommended recipe is bu yang huan wu decoction modified (Grading of recommendation: C, level of evidence: IV) [16–18].

##### Acupuncture

There is evidence demonstrating the effectiveness of acupuncture in the treating dysphagia, but a higher level of evidence-based studies is still required (Grading of recommendation: B, level of evidence: IIa) [16–18].

##### Shoulder-hand syndrome

Shoulder-hand syndrome is characterized by complex pain of the upper arm after stroke. It is considered as Yang deficiency and stasis of Blood in CM.

#### Fuming-washing therapy

Chinese herbal fuming-washing therapy and medicated bath have the effect of warming the meridian to active blood, and free the collateral vessels to expel stasis. This method can be applied directly on the local area to eliminate swelling. Apply the medical solution of fu yuan tong luo decoction directly on the hemiplegic limbs [36, 37] (Grading of recommendation: B, level of evidence: IIa).

#### Acupuncture

Principal points: Jianyu (LI 15), Jianliao (TE 14), Jianzhen (SI 9), Jianqian, (EX without international nomenclature), Ashi-point, Yemen (TE 2), Yangchi (TE 4), Wangu (SI 4) (Grading of recommendation: C, level of evidence: IV) [18].

## Conclusions

In conclusion, this is the first evidence-based CM CPG for stroke management in Hong Kong that provides the state-of-the-art clinical evidence for both herbal medicine and acupuncture. The process followed the three phases outlined in the protocol [7]. As clinical practice is often based on the practitioner’s anecdotal experience, the risk of CM application exists. Therefore, the development of CM CPGs that aim to standardize CM clinical practice and ensure its safe and effective application in Hong Kong is imperative. Involving local CM practitioners throughout the guideline development process helped ensure the CPGs are relevant to the practice of CM in Hong Kong. The next steps include disseminating the guidelines and promoting the uptake of the recommendations by Hong Kong CM practitioners.

## Guideline development panel

Aiping Lu School of Chinese Medicine, Hong Kong Baptist University.

Zhaoxiang Bian School of Chinese Medicine, Hong Kong Baptist University.

Xudong Tang Xiyuan Hospital, China Academy of Chinese Medical Sciences.

Linda L. D. Zhong School of Chinese Medicine, Hong Kong Baptist University.

Nannan Shi China Academy of Chinese Medical Sciences.

Ying Gao Department of Neurology, Dongzhimen Hospital, Beijing University of Chinese Medicine.

## Supplementary information

**Additional file 1: Appendix S1.** Clinical Practice Guidelines Development Protocol.

**Additional file 2: Appendix S2.** Details of Assessments.

**Additional file 3: Appendix S3.** Levels of Evidence and Classes of Recommendations.

## Data Availability

Details of data mining, selection, extraction and assessment carried out to support the findings of this study are available from the corresponding author upon request.

## Abbreviations

CM: Chinese Medicine
CPG: Clinical practice guideline
CACM: China Association of Chinese Medicine
CNKI: Chinese National Knowledge Infrastructure
CBM: Chinese Biomedical Literature
AGREE: The Appraisal of Guidelines for Research & Evaluation
