# Supplementary material for: Evidence-based Chinese Medicine Clinical Practice Guideline for Stroke in Hong Kong
Source: Chin Med. 2020 Nov 3;15:116. doi: 10.1186/s13020-020-00397-9 (PMC7607854; doi:10.1186/s13020-020-00397-9)
Supplement: Supplementary file 2 — Additional file 2: Appendix S2. Details of Assessments. [file 13020_2020_397_MOESM2_ESM.docx]

**Additional file 2: Appendix S2: Details of Assessments**

**A.** **Search process for seed CM CPGs**

The data from 5 databases were collected until Jan 2014 to identify the situation of the CM CPG in the past two decades. The international specific public databases for CPG including National Guideline Clearinghouse (NGC) and Guidelines International Network (G-I-N) were searched for TCM CPGs for ischemic stroke. As there is no specific public database in China for TCM CPGs, we therefore searched for TCM CPGs on the 3 public databases in China: CSSN (<http://www.cssn.net.cn>), which is the largest comprehensive standard service web site in China, SinoMed ([http://www.sinomed.ac.cn](http://www.sinomed.ac.cn/)) and CNKI (<http://www.cnli.net>), which is the main source of professional technical literature on biomedicine in China. The following search terms were used: “clinical practice guideline,” “traditional Chinese medicine”, “Stroke”, “Cerebral infarction”. The search was supplemented by manual retrievals of publications in Hong Kong and abroad.

**TABLE 1: Clinical practice guidelines in traditional Chinese medicine for stroke**

| **No** | **Title** | **Year** | **Issued by** | **Type*** | **Level** | **Dissemination** |
| --- | --- | --- | --- | --- | --- | --- |
| CPG1  [1] | Evidence-based guidelines of clinical practice in Chinese medicine internal medicine | 2011 | China Academy Of Chinese Medical Sciences | EB | Academic association level | Published book |
| CPG2  [2] | Guidelines for Diagnosis and Treatment of Common Internal Diseases in Chinese and Medicine Diseases of Modern Medicine (ZYYXH/T124-2008) | 2008 | China Association of Chinese Medicine | CB | Professional administration level | Published book |
| CPG3  [3] | Guidelines for Diagnosis and Treatment of Common Internal Diseases in Chinese Medicine Symptoms in Chinese Medicine (ZYYXH/T22-2008) | 2008 | China Association of Chinese Medicine | CB | Academic association level | Published book |
| CPG4  [4] | Guidelines for clinical research on new traditional Chinese drugs | 2002 | Ministry of Health of the People’s Republic of China | CB | Academic association level | Published book |
| CPG5  [5] | Standard for diagnosis and pattern differentiation of stroke | 1996 | Encephalopathy emergency group, SATCM | CB | Academic association level | Academic Journal |

*Type: EB = Evidence-based; CB = Consensus-based

**TABLE 2: Scopes of clinical practice guidelines in traditional Chinese medicine for stroke**

| **No** | **Title** | **Diagnostic criteria** | | **Interventions** | | | **Prevention and nurturing** | **Treatment evaluation** |
| --- | --- | --- | --- | --- | --- | --- | --- | --- |
|  |  | Classifications of TCM pattern | Diagnostic criteria of Biomedicine | Chinese formulas | Acupuncture | Other non-drug therapy |  |  |
| CPG1 | Evidence-based guidelines of clinical practice in Chinese medicine internal medicine | √ |  |  |  |  |  | √ |
| CPG2 | Guidelines for Diagnosis and Treatment of Common Internal Diseases in Chinese and Medicine Diseases of Modern Medicine (ZYYXH/T124-2008) | √ | √ | √ | √ | √ |  |  |
| CPG3 | Guidelines for Diagnosis and Treatment of Common Internal Diseases in Chinese Medicine Symptoms in Chinese Medicine (ZYYXH/T22-2008) | √ |  | √ | √ | √ | √ |  |
| CPG4 | Guidelines for clinical research on new traditional Chinese drugs | √ | √ |  |  |  |  | √ |
| CPG5 | Standard for diagnosis and pattern differentiation of stroke | √ | √ | √ | √ | √ | √ |  |

**References**

1. China Academy of Chinese Medical Sciences. Evidence-based Guidelines of Clinical Practice in Chinese Medicine Internal Medicine, Beijing:2011;1:283-310.
2. China Association of Chinese Medicine. Guidelines for Diagnosis and Treatment of Common Internal Diseases in Chinese and Medicine Diseases of Modern Medicine, Beijing:2008:260—264.
3. China Association of Chinese Medicine. Guidelines for Diagnosis and Treatment of Common Internal Diseases in Chinese Medicine Symptoms in Chinese Medicine, Beijing:2008:56—62.
4. Ministry of Health of the People’s Republic of China. Guidelines for Clinical Research on New Traditional Chinese Drugs, Beijing:2002:99-104.
5. State Administration of Traditional Chinese Medicine. Standard for Diagnosis and Pattern Differentiation of Stroke, Journal of Beijing University of TCM，1996:19,1:55-56.

**B. Recommendations**

TABLE 3: Recommendations on pattern classification in the clinical practice guidelines in traditional Chinese medicine for stroke

| Title | Patterns** | | | | | | | | | | |
| --- | --- | --- | --- | --- | --- | --- | --- | --- | --- | --- | --- |
|  | A | B | C | D | E | F | G | H | I | J | K |
| Evidence-based guideline of cerebral infarction in traditional Chinese medicine | • | • | • | • | • | • | • |  |  |  |  |
| Guideline for diagnosis and treatment of cerebral infarction in traditional Chinese medicine | • | • | • | • | • | • | • |  |  |  |  |
| Guideline for diagnosis and treatment of [stroke](app:ds:arthralgia) in traditional Chinese medicine | • | • | • | • | • | • | • | • |  |  |  |
| Guideline on cerebral infarction for clinical researches of new traditional Chinese drugs |  |  |  |  | • | • | • | • | • | • | • |
| Standard for diagnosis and effectiveness assessment of stroke in traditional Chinese medicine |  |  |  |  | • | • | • | • | • | • | • |

**** Patterns**

A= Internal obstruction of phlegm-heat pattern *(Sudden onset, loss of consciousness, nasal snore and wheezy phlegm, hemiplegia ,stiffness and spasm in the affected limb, feverish sensations in the nape and neck, restlessness, or cold extremities and convulsion, a red tongue with a yellow and greasy and dry coating and a wiry ,slippery and rapid pulse ).*

B= Phlegm misting the heart-mind pattern *(Loss of consciousness, hemiplegia, deviation of tongue and mouth, profuse sputum or salivation, a pale complexion and dark lips, incontinence of urine and stool, a dark-purple and atrophic tongue with a white and greasy coating, sunken, slippery and moderate pulse ).*

C= Declining and failure of yuan-primordial qi pattern *(Loss of consciousness, closed eyes with mouth open, paralysis and weakness of the affected limb, cold extremities, sweats, incontinence of urine and stool, a dark-purple and atrophic tongue with a white and greasy coating, extremely faint pulse).*

D= Wind phlegm obstructing the meridians pattern *(Hemiplegia, deviation of the tongue and mouth, slurred speech, numbness in the affected limb, dizziness, stick mouth with profuse sputum, dark-red tongue with a white and greasy coating, wiry and slippery pulse).*

E= Phlegm-heat in the fu-organs pattern *(Hemiplegia, stiffness and spasm of the effected limbs, slurred speech, deviation of the tongue and mouth, abdominal distension and constipation ,dizziness, blurred vision, stick mouth with profuse sputum, a red tongue with yellow and greasy coating, a wiry and slippery pulse).*

F = Syndrome of blood stasis due to qi deficiency*（Hemiplegia, paralysis and weakness of the affected limbs, deviation of the tongue and mouth, a bright pale complexion, shortness of breath, lassitude, numbness in the affected side, palpitation, spontaneous sweating, a dark tongue with a thin and white or white and greasy coating, a sunken and fine pulse).*

G= Wind-stirring due to Yin deficiency pattern (*Hemiplegia, deviation of the tongue and mouth, slurred speech, numbness in the affected limb，feverish sensations in palms, soles and chest, dizziness, blurred vision ,a red tongue with no or a scanty coating ,thready, fine and rapid pulse).*

H= Upward-disturbance of wind-fire pattern *(Hemiplegia, deviation of the tongue and mouth, slurred speech, numbness in the affected side, headache with a distending sensation, a red face and eyes ,irritability, constipation, bloody urine, a red tongue with a yellow coating, wiry and rapid pulse).*

I= Hyperactivity of wind-phlegm and fire pattern *(Hemiplegia, deviation of the tongue and mouth, slurred speech, sensory deprivation, sudden onset, dizziness, irritability, stiffness and spasm of the affected limb, stick mouth with profuse sputum, a red tongue with a yellow and greasy coating, wiry and slippery pulse).*

J= Win-phlegm and stasis obstructing the meridians pattern (*Hemiplegia, deviation of the mouth and tongue, slurred speech, sensory deprivation, dizziness, stick mouth with profuse sputum, a dark-red tongue with white thin or white and greasy coating, a wiry and slippery pulse).*

K= Phlegm-dampness misting the heart-mind pattern *(Hemiplegia, deviation of the mouth and tongue, slurred speech, sensory deprivation, loss of consciousness, wheezy phlegm, incontinence of urine and stool, a dark-purple with white and greasy coating, a deep and moderate and slippery pulse).*

TABLE 4: Recommendations on management in the clinical practice guidelines in traditional Chinese medicine for stroke

|  | CPG1 | | CPG2 | | CPG3 | | CPG4 | | CPG5 | |
| --- | --- | --- | --- | --- | --- | --- | --- | --- | --- | --- |
|  | CHM& CPM# | Rehabilitation approaches | CHM& CPM# | Rehabilitation approaches | CHM& CPM# | Rehabilitation approaches | CHM& CPM# | Rehabilitation approaches | CHM& CPM# | Rehabilitation approaches |
| Pattern A | *Ling Yang Jiao Decoction*  *An Gong Niu Huang Pills*  *Jue Fang Zhi Bao Dan*  *Niu Huang Qing Xin Pills* | Rehabilitation training  Acupuncture  Massage  Medicated bath | *Ling Yang Jiao Decoction*  *Huang Lian Jie Du Decoction*  *An Gong Niu Huang Pills*  *Jue Fang Zhi Bao Dan* | Rehabilitation training  Acupuncture  Massage  Medicated bath | *Qing Xin Xuan Qiao Decoction*  *An Gong Niu Huang Pills*  *Niu Huang Qing Xin Pills*  *Zi Xue San*  *Qing Kai Ling Injection* | Acupuncture  Massage  Medicated bath |  |  |  |  |
| Pattern B | *Di Tan Decoction*  *Su He Xiang Pills* |  | *Di Tan Decoction*  *Su He Xiang Pills* |  | *Di Tan Decoction*  *Su He Xiang Pills*  *Xing Nao Jing Injection* |  |  |  |  |  |
| Pattern C | *Shen Fu Decoction*  *Si Ni Decoction*  *Sheng Mai Yin* |  | *Shen Fu Decoction* |  | *Shen Fu Decoction*  *Sheng Mai San*  *Shen Fu Injection*  *Shen Mai Injection* |  |  |  |  |  |
| Pattern D | Hua Tan Tong Luo Decoction  Tong Mai Capsules  Xin Mai Tong Capsules |  | *Hua Tan Tong Luo Decoction* |  | *Hua Tan Tong Luo Decoction*  *Quan Tian Ma Capsules*  *Zhong Feng Hui Chun Pills* |  |  |  |  |  |
| Pattern E | *Xing Lou Cheng Qi Decoction*  *Da Cheng Qi Decoction*  *Da Chai Hu Decoction* |  | *Xing Lou Cheng Qi Decoction*  *Di Dang Decoction* |  | *Xing Lou Cheng Qi Decoction*  *Da Chai Hu Decoction*  *Xin Qing Ning Pian*  *Niu Huang Qing Xin Pills*  *Qing Kai Ling Injection* |  |  |  |  |  |
| Pattern F | *Bu Yang Huan Wu Decoction*  *Nao Xin Tong Capsules* |  | *Bu Yang Huan Wu Decoction* |  | *Bu Yang Huan Wu Decoction*  *Nao Xin Tong Capsules*  *Nao An Capsules*  *Xiao Shuan Tong Luo Pian*  *Sheng Mai Injection* |  |  |  |  |  |
| Pattern G | *Zhen Gan Xi Feng Decoction*  *Yu Yin Xi Feng Decoction* |  | *Yu yin Tong Luo Decoction* |  | *Yu Yin Tong Luo Decoction*  *Da Bu Yin Pills*  *Tian Ma Gou Teng*  *Granules* |  |  |  |  |  |
| Pattern H |  |  | *Tian Ma Tou Teng Yin* |  |  |  |  |  |  |  |

**#** **CHM&CPM = Chinese Herbal Medicine & Chinese Proprietary Medicine**

- ***Ling Yang Jiao Decoction*** *(*Ingredients*: Saigae Tataricae Cornu (Ling Yang Jiao Fen), Haliotidis Concha (Sheng Shi Jue Ming), Prunellae Spica (Xia Ku Cao), Chrysanthemi Flos (Ju Hua), Testudinis Carapax Et Plastrum (Gui Ban), Rehmanniae Radix (Sheng Di), Moutan Cortex (Mu Dan Pi), Paeoniae Radix Alba (Bai Shao), Bambusae Concretio Silicea (Tian Zhu Huang), Arisaematis Rhizoma (Zhi Nan Xing)).*
- ***Huang Lian Jie Du*** ***Decoction*** *(Ingredients: Coptidis Rhizoma (Huang Lian), Scutellariae Radix (Huang Qin), Phellodendri Chinensis Cortex (Huang Bo), Gardeniae Fructus (Zhi Zi)).*
- ***An Gong Niu Huang Tablets*** *(*Ingredients*: Bovis Calculus (Niu Huang), Bubali Cornu (Shui Niu Jiao), Moschus (She Xiang), Margarita (Zhen Zhu), Realgar (Xiong Huang), Cinnabaris (Zhu Sha), Coptidis Rhizoma (Huang Lian), Scutellariae Radix (Huang Qin), Gardeniae Fructus (Zhi Zi), Curcumae Radix (Yu Jin), Borneolum Syntheticum (Bin Pian)).*
- ***Ju Fang Zhi Bao Boluses*** *(*Ingredients*: Bubali Cornu (Shui Niu Jiao), Bovis Calculus (Niu Huang), Hawksbill Turtle (Dai Mao), Moschus (She Xiang), Cinnabaris (Zhu Sha), Ambrum (Hu Po), Benzoinum (An Xi Xiang), Realgar (Xiong Huang), Borneolum Syntheticum (Bin Pian)).*
- ***Niu Huang Qing Xin Tablets*** *(*Ingredients*: Bovis Calculus (Niu Huang), Angelicae Radix (Dang Gui)and 27 herbs)).*
- ***Qing Xin Xuan Qiao Decoction (***Ingredients*: Coptidis Rhizoma (Huang Lian), Salviae Miltiorrhizae Radix Et Rhizoma (Dan Shen), Gastrodiae Rhizoma (Tian Ma), Uncariae Ramulus Cum Uncis (Gou Teng), Acori Tararinowii Rhizoma (Shi Chang Pu), Moutan Cortex (Mu Dan Pi), Saigae Tataricae Cornu (Ling Yang Jiao Fen)).*
- ***Qing Kai Ling Injection (***Ingredients*: Margaritifera Concha (Zhen Zhu Mu) and 8 herbs****).***
- ***Di Tan Decoction (***Ingredients*: Pinelliae Rhizoma Praeparatum (Fa Ban Xia), Citri Reticulatae Pericarpium (Chen Pi), Aurantii Fructus Immaturus (Zhi Shi), Arisaema Cum Bile (Dan Nan Xing), Poria (Fu Ling), Acori Tararinowii Rhizoma (Shi Chang Pu), Bambusae Caulis In Taenias (Zhu Ru), Polygalae Radix (Yuan Zhi), Salviae Miltiorrhizae Radix Et Rhizoma (Dan Shen), Glycyrrhizae Radix Et Rhizoma (Gan Cao)).*
- ***Su He Xiang Pills (***Ingredients*: Styrax (Su He Xiang), Benzoinum (An Xi Xiang), Borneolum Syntheticum (Bin Pian), Bubali Cornu (Shui Niu Jiao),* *Moschus (She Xiang),* *Lignum Santali Albi (Tan Xiang),* *Aquilaria agallocha Roxb (Chen Xiang),* *Syzygium Aromaticum (Ding Xiang),* *Rhizoma Cyperi (Xiang Fu),* *Radix Aucklandiae (Mu Xiang),* *Olibanum (Ru Xiang),* *Fructus Piperis Longi (Bi Ba),* *Rhizoma Atractylodis Macrocephalae (Bai Zhu), Chebulae Fructus (He Zi), Cinnabaris (Zhu Sha)).*
- ***Xing Nao Jing Injection*** ***(***Ingredients*:* *Moschus (She Xiang), Curcumae Radix (Yu Jin), Borneolum Syntheticum (Bin Pian), Gardeniae Fructus (Zhi Zi))*
- ***Shen Fu Decoction (***Ingredients*: Ginseng Radix Et Rhizoma (Ren Shen), Aconiti Lateralis Radix Praeparata (Fu Zi)).*
- ***Si Ni Decoction (***Ingredients*: Bupleuri Radix (Chai Hu), Paeoniae Radix Alba (Bai Shao), Aurantii Fructus Immaturus (Zhi Shi), Glycyrrhizae Radix Et Rhizoma (Gan Cao)).*
- ***Sheng Mai Yin (***Ingredients*: Ginseng Radix Et Rhizoma (Ren Shen), Ophiopogonis Radix (Mai Dong), Schisandrae Chinensis Fructus (Wu Wei Zi)).*
- ***Sheng Fu Injection*** ***(***Ingredients: *Ginseng Radix Et Rhizoma (Ren Shen), Aconiti Lateralis Radix Praeparata (Fu Zi)).*
- ***Sheng Mai Injection (***Ingredients*: Ginseng Radix Et Rhizoma (Ren Shen), Ophiopogonis Radix (Mai Dong)).*
- ***Hua Tan Tong Luo Decoction (***Ingredients*: Pinelliae Rhizoma Praeparatum (Fa Ban Xia), Atractylodis Macrocephalae Rhizoma (Bai Zhu), Gastrodiae Rhizoma(Tian Ma), Salviae Miltiorrhizae Radix Et Rhizoma (Dan Shen), Rhizoma Cyperi (Xiang Fu), Arisaema Cum Bile (Dan Nan Xing), Rhei Radix Et Rhizoma (Da Huang)).*
- ***Tong Mai Capsules (***Ingredients: *Salviae Miltiorrhizae Radix Et Rhizoma (Dan Shen), Chuanxiong Rhizoma (Chuan Xiong), Puerariae Lobatae Radix (Ge Gen)).*
- ***Xin Mai Di Capsules (***Ingredients*: Angelicae Sinensis Radix (Dang Gui),* *Salviae Miltiorrhizae Radix Et Rhizoma (Dan Shen), Puerariae Lobatae Radix (Ge Gen), Cyathulae Radix (Chuan Niu Xi), Uncariae Ramulus Cum Uncis (Gou Teng), Sophorae Flos (Huai Hua), Cassiae Semen (Jue Ming Zi), Notoginseng Radix Et Rhizoma (San Qi), Prunellae Spica (Xia Ku Cao)).*
- ***Quan Tian Ma Capsules (***Ingredients*: Gastrodiae Rhizoma (Tian Ma)).*
- ***Zhong Feng Hui Chun Pills (***Ingredients: *Angelicae Sinensis Radix (Dang Gui), Chuanxiong Rhizoma (Chuang Xiong), Carthami Flos (Hong Hua)and 15 herbs.*
- ***Xing Lou Cheng Qi Decoction (***Ingredients: *Trichosanthis Fructus (Gua Lou), Arisaema Cum Bile (Dan Nan Xing), Rhei Radix Et Rhizoma (Da Huang), Natrii Sulfas (Mang Xiao)).*
- ***Da Cheng Qi Decoction (***Ingredients: *Rhei Radix Et Rhizoma (Da Huang), Natrii Sulfas (Mang Xiao), Aurantii Fructus Immaturus (Zhi Shi), Magnoliae Officinalis Cortex (Hou Po)).*
- ***Da Chai Hu Decoction (***Ingredients: *Bupleuri Radix (Chai Hu), Paeoniae Radix Alba (Bai Shao), Aurantii Fructus Immaturus (Zhi Shi), Scutellariae Radix (Huang Qin), Glycyrrhizae Radix Et Rhizoma (Gan Cao)).*
- ***Di Dang Decoction (***Ingredients*: Hirudo (Shui Zhi), Tabanus (Meng Chong), Persicae Semen (Tao Ren), Rhei Radix Et Rhizoma (Da Huang)).*
- ***Xin Qing Ning Pian (***Ingredients: *Rhei Radix Et Rhizoma (Da Huang)).*
- ***Bu Yang Huan Wu Decoction (***Ingredients: *Astragali Radix (Huang Qi), Angelicae Sinensis Radix (Dang Gui),* *Persicae Semen (Tao Ren),* *Carthami Flos (Hong Hua),* *Chuanxiong Rhizoma (Chuang Xiong), Pheretima (Di Long), Paeoniae Radix Rubra (Chi Shao)).*
- ***Nao Xin Tong Capsules (***Ingredients*: Astragali Radix (Huang Qi), Paeoniae Radix Rubra (Chi Shao)*, *Salviae Miltiorrhizae Radix Et Rhizoma (Dan Shen), Angelicae Sinensis Radix (Dang Gui), Chuanxiong Rhizoma (Chuan Xiong), Persicae Semen (Tao Ren), Carthami Flos (Hong Hua), Olibanum (Ru Xiang), Myrrha (Mo Yao), SpatholobiCaulis (Ji Xue Teng), Achyranthis Bidentatae Radix (Niu Xi), Persicae Ramulus (Gui Zhi), Scorpio (Quan Xie), Pheretima (Di Long), Mori Ramulus (Sang Zhi),Hirudo (Shui Zhi)).*
- ***Tong Xin Luo Capsules (***Ingredients: *Ginseng Radix Et Rhizoma (Ren Shen), Hirudo (Shui Zhi), Scorpio (Quan Xie), Paeoniae Radix Rubra (Chi Shao), Cicadae Periostracum (Chan Tui), Eupolyphaga (Tu Bie Chong), Scolopendra (Wu Gong), Lignum Santali Albi (Tan Xiang), Olibanum (Ru Xiang), Dalbergiae Odoriferae Lignum (Jiang Xiang), Ziziphi Spinosae Semen (Suan Zao Ren)).*
- ***Nao An Capsules (***Ingredients: *Ginseng Radix Et Rhizoma (Ren Shen), Chuanxiong Rhizoma (Chuan Xiong), Angelicae Sinensis Radix (Dang Gui), Carthami Flos* (*Hong Hua), Borneolum Syntheticum (Bin Pian)).*
- ***Xiao Shuang Tong Luo Pian (***Ingredients*: Chuanxiong Rhizoma (Chuan Xiong), Salviae Miltiorrhizae Radix Et Rhizoma (Dan Shen), Astragali Radix (Huang Qi), Alismatis Rhizoma (Ze Xie), Notoginseng Radix Et Rhizoma (San Qi),Sophorae Flos (Huai Hua), Cinnamoni Ramulus (Gui Zhi), Curcumae Radix (Yu Jin), Borneolum Syntheticum (Bin Pian), Crataegi Fructus (Shan Zha), OsDraconis (Sheng Long Gu)).*
- ***Sheng Mai Injection (***Ingredients: *Ginseng Radix Et Rhizoma Rubra (Hong Shen), Ophiopogonis Radix (Mai Dong), Schisandrae Chinensis Fructus (Wu Wei Zi)*
- ***Zhen Gan Xi Feng Decoction (***Ingredients: *Achyranthis Bidentatae Radix (Huai Niu Xi), Haematitum (Zhe Shi), Ostreae Concha (Sheng Mu Li), Testudinis Carapax Et Plastrum (Gui Ban), Paeoniae Radix Alba (Bai Shao), Scrophulariae Radix (Xuan Shen), Asparagi Radix (Tian Dong), Toosendan Fructus (Chuan Lian Zi), Hordei Fructus Germinatus (Sheng Mai Ya), Artemisiae Scopariae Herba (Yin Chen), Glycyrrhizae Radix et Rhizoma (Gan Cao)).*
- ***Yu Yin Xi Feng Decoction (***Ingredients: *Rehmanniae Radix (Sheng Di Huang), CorniFructus (Shan Zhu Yu), Uncariae Ramulus Cum Uncis(Gou Teng), Gastrodiae Rhizoma(Tian Ma), Salviae Miltiorrhizae Radix Et Rhizoma (Dan Shen), Paeoniae Radix Alba(Bai Shao)).*
- ***Da Bu Yin Pills (***Ingredients: *Rehmanniae Radix Praeparata* (*Shu Di Huang), Anemarrhenae Rhizoma (Zhi Mu), Phellodendri Chinensis Cortex (Huang Bo), Testudinis Carapax Et Plastrum (Gui Ban)).*
- ***Tian Ma Gou Teng Yin (***Ingredients: *Gastrodiae Rhizoma (Tian Ma), Uncariae Ramulus Cum Uncis (Gou Teng), Haliotidis Concha (Sheng Shi Jue Ming), Cyathulae Radix (Chuan Niu Xi), Scutellariae Radix (Huang Qin), Gardeniae Fructus (Zhi Zi), Prunellae Spica (Xia Ku Cao)).*

**C. AGREE II tool appraisals of 5 CM CPGs for stroke**

**Figure 1. Average domain score of the clinical practice guidelines in traditional Chinese medicine for stroke according to the AGREEII instrument**

**Figure 2. Domains score of the clinical practice guidelines in traditional Chinese medicine for stroke according to the AGREEII instrument**
