# Supplementary material for: Evidence-based Chinese Medicine Clinical Practice Guideline for Stroke in Hong Kong
Source: Chin Med. 2020 Nov 3;15:116. doi: 10.1186/s13020-020-00397-9 (PMC7607854; doi:10.1186/s13020-020-00397-9)
Supplement: Supplementary file 3 — Additional file 3: Appendix S3. Levels of Evidence and Classes of Recommendations. [file 13020_2020_397_MOESM3_ESM.docx]

**Additional file 3: Appendix S3: Levels of Evidence and Classes of Recommendations**

**Level of evidence [1]**

Utilize the evidence grading systems for CM established by Prof. Liu Jianping from China:

Class Ⅰa: Data derived from at least two different types of these four research methods: randomized controlled clinical trial, cohort study, case controlled study or case series study, and consistent across the different studies;

Class Ⅰb: Data derived from a single randomized controlled clinical trial with sufficient power;

Class Ⅱa: Data derived from randomized controlled clinical trials and cohort studies;

Class Ⅱb: Data derived from case controlled study;

Class Ⅲa: Data derived from historical controlled case series studies;

Class Ⅲb: Data derived from self-controlled case series studies;

Class Ⅳ: Data derived from widely-used clinical case reports and the historical records of therapy for long time;

Class Ⅴ: Data derived from experts’ opinion and clinical trials without systemic researches; Data derived from case reports and the historical records of therapy without wide and long-term usage clinically.

**Grading of recommendations [1]**

Utilize and revise appropriately the recommendation level systems established by American National Guideline Clearinghouse.

Level A: It should contain at least one randomized controlled clinical trial which was high-quality and provided specific suggestion consistently in a part of the literature (Evidence from Ⅰa and Ⅰb);

Level B: It needed to contain theme-related well-achieved clinical trials but was absent of randomized controlled clinical trials (Evidence from Ⅱa, Ⅱb and Ⅲ);

Level C: It needed to contain reports, opinion, and/or clinical experience from experts committee, but was absent of high-quality clinical trials (Evidence from Ⅳ and Ⅴ).

**Reference:**

1. Liu JP. The composition of evidence body of traditional medicine and recommendations for its evidence grading. Zhongguo Zhong Xi Yi Jie He Za Zhi. 2007; 27(12): 1061-1065.
